# P2X7 Receptor-Induced Human Mast Cell Degranulation Is Enhanced by Interleukin 33

**DOI:** 10.3390/ijms25031730

**Published:** 2024-01-31

**Authors:** Barbora Salcman, Rajia Bahri, Peter W. West, Chiara Tontini, Karen Affleck, Silvia Bulfone-Paus

**Affiliations:** 1 Lydia Becker Institute of Immunology and Inflammation, University of Manchester, Manchester M13 9NT, UK; barbora.salcman@manchester.ac.uk (B.S.); rajia.bahri-2@manchester.ac.uk (R.B.); peter.west@manchester.ac.uk (P.W.W.); chiara.tontini@postgrad.manchester.ac.uk (C.T.); 2 GlaxoSmithKline, Stevenage SG1 2NY, UK; karen.x.affleck@gsk.com

**Keywords:** mast cells, IL-33, ATP, P2X1, P2X4, P2X7

## Abstract

MCs are tissue-resident immune cells that strategically reside in barrier organs and respond effectively to a wide range of stimuli, such as IL-33, a mediator released upon epithelial damage. Adenosine triphosphate (ATP) accumulates at sites of tissue injury and is known to modulate MC activities. This study investigated how an inflammatory tissue environment rich in IL-33 modulates the ATP-mediated activation of MCs. Human primary MCs primed with IL-33 displayed a strongly increased response to ATP but not ADP. This resulted in increased degranulation, IL-8 release, and pERK1/2 signalling. Such effects are unique to IL-33 stimulation and not shared by the epithelial alarmin, TSLP. MC exposure to IL-33 also increased membrane expression of purinergic and ATP-binding P2X receptors. The use of selective P2X receptor inhibitors identified P2X7 receptor as the key mediator of the enhanced ATP-induced ERK1/2 signalling and degranulation in IL-33-primed MCs. Whilst the inhibition of P2X1 and P2X4 receptors had no effect on MC degranulation, inhibiting these receptors together with P2X7 resulted in further decreased MC-mediated degranulation. These data therefore point toward the potential mechanisms by which IL-33 contributes to the modulation of ATP-mediated activation in human MCs.

## 1. Introduction

Mast cells (MCs) display a broad spectrum of receptors, through which they respond to several exogenous and endogenous mediators, resulting in the release of cytokines, chemokines, growth factors, and/or lipid mediators [1,2]. IgE and anti-IgE, the complement system, Toll-like receptor ligands, and adenosine triphosphate (ATP) are well known inducers of MC activation and degranulation [3,4].

ATP, a purine nucleotide fundamental to almost all biological functions and the ultimate source of energy for cells, is involved in a plethora of metabolic and non-metabolic functions [5]. At low concentrations (about 10 nM), extracellular ATP, or ATP contained in vesicles, serves as a neurotransmitter [6]. In inflammatory conditions, epithelial and endothelial cells release ATP through various means, such as exocytosis, non-specific release, or through ion channels such as pannexin [7,8,9]. In healthy tissue, extracellular ATP is hydrolysed stepwise by CD39 and CD73 to ADP, AMP, and adenosine [10,11]. Both ATP and its metabolites are key signalling molecules involved in inflammasome activation and inflammatory cytokine secretion [12].

ATP and ADP can activate purinergic receptors, and ATP is able to activate all seven members of the P2X receptor family (P2X1, P2X2, P2X3, P2X4, P2X5, P2X6, P2X7) and some P2Y receptors (P2Y2, P2Y11, P2Y12), while ADP can only activate specific P2Y receptors (P2Y1, P2Y12, P2Y13) [13,14]. Only three members of the P2X receptor family—P2X1, P2X4, and P2X7—are expressed and functional in MCs [15,16]. Of these, P2X7 is the main receptor responsible for ATP-induced MC degranulation [16,17]. Conversely, neither P2X1 receptor activation nor P2X4 receptor activation induce MC degranulation, although P2X4 engagement can significantly increase IgE-mediated degranulation in mice [17,18]. All three P2X receptors expressed by MCs promote calcium influx and the activation of intracellular signalling pathways. The ATP affinity of each receptor differs greatly, ranging from sub-micromolar concentrations for P2X1 receptor to low micromolar concentrations for P2X4 receptor. In contrast, P2X7 requires ATP concentrations above 100 µM for its activation [15].

Upon tissue trauma, a wide range of alarmins are released from epithelial and endothelial cells, among which the most notable is IL-33, a member of the IL-1 family that was first described by Schmitz et al. [19]. Since its discovery, IL-33 has been shown to influence several inflammatory processes and has been linked to several pathological conditions [20,21]. In MCs, IL-33 promotes a wide range of functions. While IL-33 itself does not induce degranulation, it promotes the release of several mediators, such as TNF-α, IL-6, MCP-1, IL-13, and IL-5 [22]. IL-33 also potentiates IgE- and complement-mediated degranulation and cytokine production, worsening inflammatory conditions and increasing the recruitment of immune cells to the site of inflammation [23]. Conversely, prolonged exposure to IL-33 leads to a decrease in FcεRI receptor expression and IgE-mediated activation in MCs [24].

Exposure to ATP itself promotes the release of IL-33 by MCs and other cell types, such as dendritic cells, keratinocytes, astrocytes, and human bronchial epithelial cells [25,26,27,28,29]. Jordan et al. [30] reported that the co-sensing of ATP and IL-33 potentiates the release of specific cytokines from mouse bone marrow-derived MCs (BMMCs), boosting COX1/2 activation and prolonging the activation of the TAK1-IKK2-NF-κB signalling pathway, which results in the production of pro-inflammatory cytokines (i.e., IL-2, IL-4, IL-6, and GM-CSF), prostaglandins, and thromboxanes. Although a clear link between MC, IL-33, and ATP in human diseases has not yet been defined, the activation of the TAK1-IKK2-NF-κB pathway is known to play a role in cancer and autoimmune diseases such as psoriasis and rheumatoid arthritis [31], diseases in which MC contribution has been investigated [32,33,34]. Straus et al. [35] observed a 3–6-fold increase in the release of IL-6, TNF-α, and IL-13 in response to ATP in BMMCs and peritoneal MCs previously sensitized with IL-33.

The aim of our study was to investigate the effect of IL-33 priming on ATP-mediated MCs activities using primary blood-derived human MCs as a model and to dissect the contribution of the receptors involved. Our findings demonstrate an IL-33-driven enhancement of ATP- but not ADP-mediated degranulation, cytokine secretion, and signalling in human MCs that relied on P2X7 engagement. Furthermore, we suggest that an IL-33-dependent microenvironment amplifies the effects of extracellular ATP, whereas the hydrolysis of extracellular ATP prevents excessive MC activities.

## 2. Results

### 2.1. Human Primary Mast Cells Degranulate upon ATP but Not ADP Stimulation

**Figure 1:**
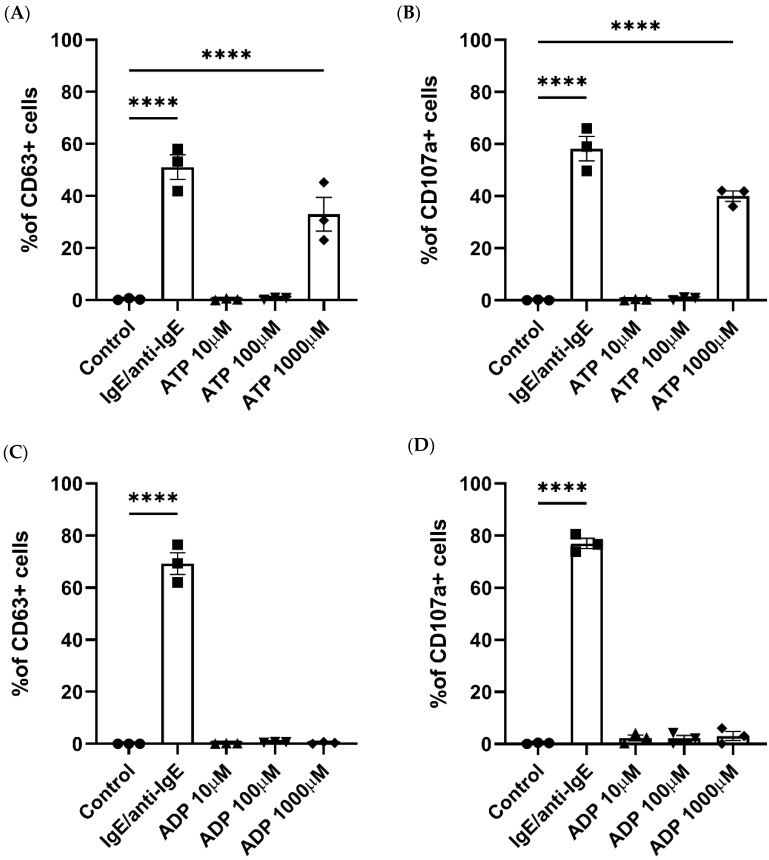
Human mast cell degranulation is induced by ATP stimulation. Degranulation in response to ATP (**A**,**B**), ADP (**C**,**D**), IgE and anti-IgE (positive control), or a negative control was measured by the externalization of CD63 (**A**,**C**) or CD107a (**B**,**D**) and analysed using flow cytometry. Data are mean ± SEM of *n* = 3 experiments from individual MC cultures. Statistical differences are indicated; **** *p* < 0.0001 (ordinary one-way ANOVA with Šídák’s post hoc test).

Formerly, LAD2 MC models have shown that ATP concentrations over 300 μM are able to induce MC degranulation [16], while ADP stimulation resulted in low-level degranulation in rat MCs [36]. We therefore investigated whether ATP and ADP can elicit similar responses in blood-derived human primary MCs. The MC gating strategy is shown in Figure S1A. Of the three ATP concentrations studied, only 1000 μM ATP resulted in a significant increase in MC degranulation, measured through the externalization of CD63 and CD107a [37], (Figure 1A,B) compared to unstimulated cells. IgE/anti-IgE stimulation was used as a positive control in all the MC activation experiments.

ADP stimulation did not produce a significant increase in MC degranulation at any of the concentrations studied (Figure 1C,D). Therefore, we confirmed that ATP can trigger degranulation in human primary MCs, while ADP stimulation exerts no effect.

### 2.2. IL-33 Priming Modulates ATP-Induced Mast Cell Activities

**Figure 2:**
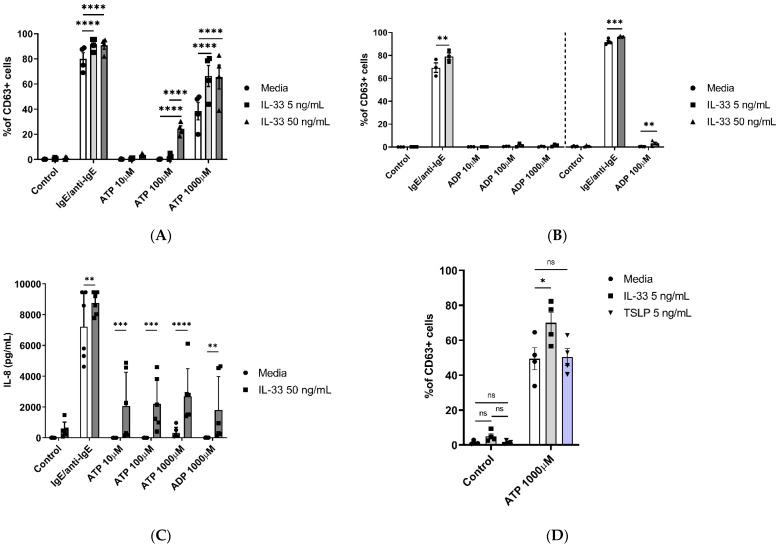
IL-33 enhances ATP-mediated MC activities. MCs were pre-treated for 24 h with media control or IL-33 at the concentrations indicated, followed by activation with IgE/anti-IgE, ATP, or a negative control (media). (**A**) Degranulation was measured by cell staining with an anti-CD63 antibody (*n* = 3 separate experiments from separate MC cultures); (**B**) effect of treatment with IL-33 5 ng/mL (*n* = 3 experiments from separate MC cultures, left) and 50 ng/mL (*n* = 3 experiments from separate MC cultures, right) on ADP-induced MC degranulation measured by anti-CD63 antibody staining. (**C**) IL-8 cytokine secretion by IL-33-treated cells stimulated with ATP and ADP for 8 h (*n* = 6 independent experiments from six individual MC cultures). (**D**) Comparison of IL-33 and TSLP pre-treatments on ATP-induced cell degranulation as measured by CD63 flow cytometry staining (*n* = 4 separate experiments in separate MC cultures). Data are mean ± SEM. Statistical differences are indicated; ns: not significant, * *p* < 0.05, ** *p* < 0.01, *** *p* < 0.001, **** *p* < 0.0001 (two-way ANOVA with Šídák’s post hoc test).

Previously, IL-33 has been shown to not only regulate key MC activities such as cell adhesion, survival, or proliferation but also play an important role in potentiating mediator release, intracellular signalling, and degranulation induced through CD117, FcεRI, or complement receptors [22]. The next step was to investigate whether IL-33 priming exerted any significant effect on promoting ATP- or ADP-induced MC degranulation. Compared to untreated cells, IL-33 priming resulted in the significant potentiation of ATP-mediated MC degranulation at a concentration of 100 and 1000 μM (Figure 2A and Figure S1B).

Under the same conditions, when IL-33 was used at 5 ng/mL concentrations with ADP stimulation, no potentiation of MC degranulation was observed for any of the ADP concentrations tested (Figure 2B). However, when using 50 ng/mL IL-33 priming, ADP stimulation at 100 μM resulted in significantly increased activation, albeit not to the same magnitude as ATP-mediated stimulation.

Since we observed IL-33 influencing ATP responses, we subsequently investigated its influence on the release of IL-8 by priming MCs for 24 h with 50 ng/mL IL-33, followed by stimulation with different concentrations of ATP (10–1000 μM) and ADP (1000 µM) for 8 h (Figure 2C). IL-33 priming significantly increased the release of IL-8 for all ATP- and ADP-stimulated cells compared to the untreated cells. These results further confirm the enhancing effect of IL-33 pre-treatment on ATP-mediated MC activities.

Upon epithelial cell damage, several different mediators are released in the extracellular compartment. One of the released alarmins, TSLP, shares similar properties with IL-33 and has been shown to promote allergic inflammation and influence MC activities [38]. We therefore questioned if the effect of IL-33 priming on ATP-mediated MC activities is also shared by TSLP. MCs were primed for 24 h with either IL-33 or TSLP 5 ng/mL, and then we stimulated the cells using ATP (1000 μM) (Figure 2D). Similar to the previously obtained data, IL-33 priming increased ATP-mediated MC degranulation when compared to the untreated cells, while TSLP priming did not. These findings point toward the distinct modulatory properties of IL-33 in influencing ATP-mediated activities in MCs.

### 2.3. IL-33 Priming Modulates the Expression of P2X Receptors

**Figure 3:**
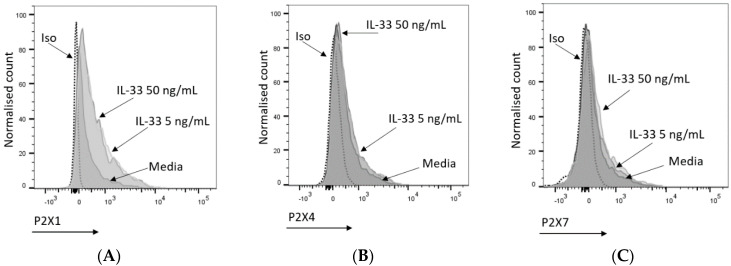

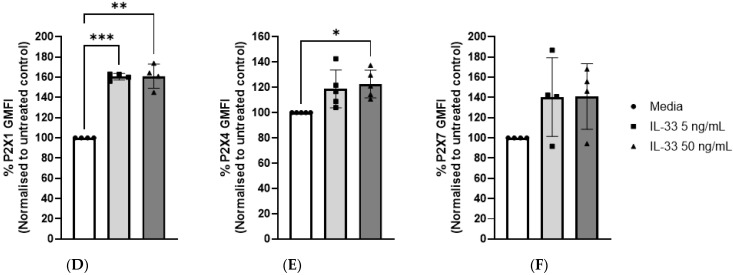
Mast cell P2X receptor expression and modulation by IL-33. (**A**–**C**) Representative histogram indicating P2X1 (**A**), P2X4 (**B**), and P2X7 (**C**) expression upon treatment with media (5 or 50 ng/mL IL-33 for 24 h). P2X1, P2X4, and P2X7 were stained and analysed by flow cytometry. The dotted lines indicate cell staining with isotype control antibodies. (**D**–**F**) IL-33-treated or untreated MCs were stained for P2X1 (*n* = 2), P2X4 (*n* = 5), and P2X7 (*n* = 4); the geometric mean of fluorescence intensity (GMFI) was normalized to the negative control (untreated samples). Data are displayed as mean ± SEM. Statistical differences are indicated by * *p* < 0.05 and ** *p* < 0.01, *** *p* < 0.001 (one-way ANOVA with Šídák’s post hoc test).

Of the seven P2XRs known to be engaged by ATP, only three are functional in MCs, namely P2X1, P2X4, and P2X7 receptors [15]. Previously, P2X7 has been shown to play an important role in MC degranulation and activation [16]. The increased susceptibility of the IL-33-pre-treated MCs to degranulation could be the result of an increased availability of P2XRs at the cell membrane. To test this hypothesis, P2X1, P2X4, and P2X7 receptor expression was measured according to geometric mean fluorescence intensity (GMFI) by flow cytometry in MCs treated with IL-33 for 24 h and conducting a comparative analysis with untreated cells. As shown in Figure 3, IL-33 priming resulted in significantly increased expression of P2X1 at both IL-33 concentrations, while significant upregulation in P2X4 expression was observed only at the IL-33 concentration of 50 ng/mL. However, the magnitude of the increase was lower than for P2X1. Conversely, P2X7 receptor expression showed great variability among donors. When a transcriptomic analysis of IL-33 priming was conducted on the MCs (Figures S2 and S3), IL-33 50 ng/mL had little effect on the transcript levels of the P2XRs expressed by the MCs. While P2RX7 transcription was significantly decreased, it was still at a high level. Exposure to IL-33 did increase transcripts from genes such as CXCL8, IL-5, or IL-13. This suggests that the effect of IL-33 priming on P2XR expression is likely to occur at a post-translational level over the timeframe studied here.

### 2.4. P2X7 Orthosteric Inhibitors Do Not Fully Inhibit the IL-33 Enhancing Effect on ATP-Mediated Degranulation

**Figure 4:**
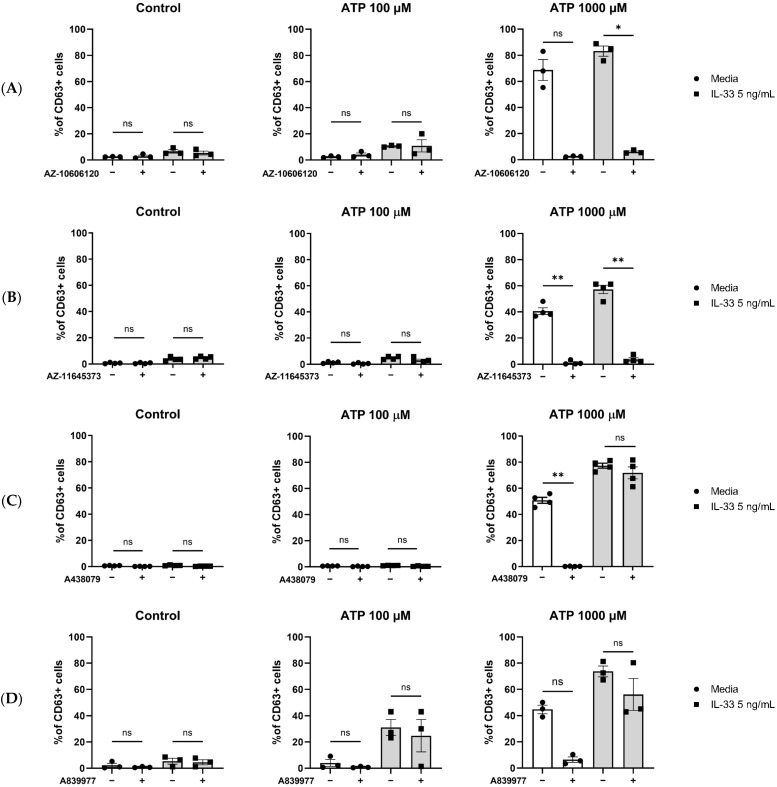
Inhibition of P2X7 receptor by orthosteric and allosteric ligands in IL-33-treated mast cells. MCs were left untreated or incubated with IL-33 for 24 h at 5 ng/mL. The MCs were then exposed to 5 μM concentration of the allosteric P2X7 inhibitors AZ-10606120 ((**A**), *n* = 4) and AZ-11645373 ((**B**), *n* = 4) and 5 μM concentration of the orthosteric P2X7 inhibitors A438079 ((**C**), *n* = 3) and A804598 ((**D**), *n* = 3) for 15 min before subsequent ATP stimulation. Data are mean ± SEM. Statistical differences are indicated as follows: ns = no significance; * *p* < 0.05; ** *p* < 0.01 (one-way ANOVA with Šídák’s post hoc test).

To investigate the contribution of the P2X7 receptor to ATP-mediated MC degranulation in general and more specifically to the potentiating effect given by IL-33 pre-treatment, P2X7 inhibitors were tested in our MC degranulation assay. MCs were primed with IL-33, incubated with or without orthosteric or allosteric inhibitors, and subsequently stimulated with ATP. The allosteric inhibitor AZ-11645373 (Figure 4A) led to a significant decrease in MC degranulation, independent of the IL-33 priming concentration and the concentrations of ATP used. Similar results were obtained using an additional allosteric inhibitor, AZ-10606120 (Figure 4B). Conversely, the use of two orthosteric P2X7 inhibitors—A438079 (Figure 4C) and A804598 (Figure 4D)—led to a decrease in degranulation only in the MCs which were not pre-treated before ATP stimulation.

Hence, we showed that P2X7 engagement contributes to ATP-mediated MC degranulation. However, P2X7 orthosteric inhibitors do not fully inhibit the IL-33 enhancing effect on ATP-induced degranulation.

### 2.5. IL-33 Priming Affects P2X7-Mediated pERK1/2 Signalling

**Figure 5:**
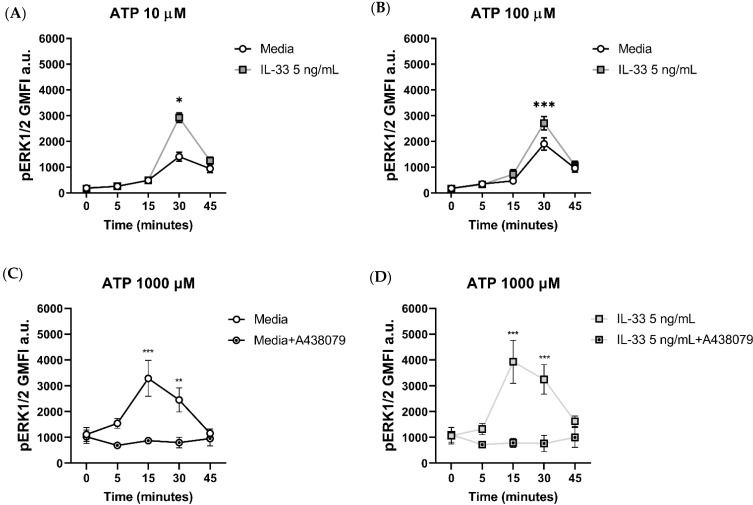
Effect of the orthosteric P2X7 inhibitor on ERK 1/2 phosphorylation in IL-33-treated MCs stimulated with ATP. MCs were left untreated or incubated with IL-33 for 24 h at the concentrations indicated and stimulated with ATP concentrations of 10 µM (**A**) and 100 µM (**B**). ERK1/2 phosphorylation was measured flow cytometry using the geometric mean of fluorescence intensity (GMFI, *n* = 3 separate experiments in separate MC cultures). (**C**,**D**) The A438079 P2X7 inhibitor was added at a concentration of 5 μM to untreated (**C**) or IL-33-treated MCs (**D**) 15 min prior to stimulation with ATP 1000 µM (*n* = 4 separate experiments from individual MC cultures). Data are mean ± SEM. Statistical differences are indicated as follows: * *p* < 0.05, ** *p* < 0.01, *** *p* < 0.001 (two-way ANOVA with Sidak’s post hoc test).

To further characterize the effect of IL-33 priming on ATP-mediated MC activation, we investigated the changes in pERK1/2 signalling upon stimulation. MCs were primed with IL-33 and then stimulated with ATP for up to 45 min. When using 10 μM ATP (Figure 5A), pERK1/2 signalling peaked at 30 min post-stimulation, with 5 ng/mL IL-33 significantly boosting pERK1/2 signalling compared to the control cells. Increased pERK1/2 signalling was also observed when MCs were exposed to IL-33 and stimulated with 100 μM ATP (Figure 5B).

To gain more insight in the dynamics of pERK1/2 signalling upon P2X7 engagement, we stimulated MCs with 1000 μM ATP for 0, 5, 15, 30, and 45 min with or without the addition of the orthosteric inhibitor A438079 prior to stimulation (Figure 5C,D). Compared to lower ATP concentrations, stimulation with ATP 1000 μM resulted in a faster increase in pERK1/2 signalling, reaching the peak at 15 min post-stimulation. The addition of the orthosteric inhibitor significantly abated pERK1/2 signalling at 15 and 30 min post-stimulation compared to the untreated MCs, regardless of the IL-33 priming concentration.

Our findings suggest that the enhanced pERK1/2 signalling mediated by IL-33 priming is linked to ATP successfully ligating the P2X7 receptor.

### 2.6. ATP-Mediated Degranulation in Human Mast Cells Does Not Require P2X1 and P2X4 Receptor Engagement

**Figure 6:**
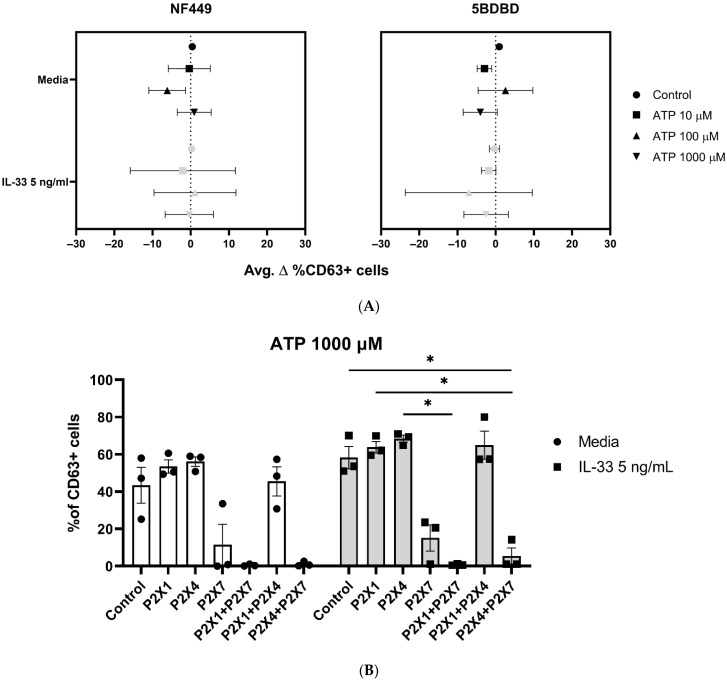
The use of P2X1 and P2X4 receptors, alone or in combination, does not affect MC degranulation. MCs were left untreated or incubated with 5 ng/mL IL-33 for 24 h. (**A**) The MCs were exposed to NF449 (P2X1 inhibitor) and 5BDBD (P2X4 inhibitor) for 15 min before ATP stimulation at the concentrations indicated (*n* = 3 separate experiments). A statistical analysis showed no significance between the control, stimulated, and IL-33-primed cells. (**B**) The MCs were exposed to NF449 (P2X1 inhibitor), 5BDBD (P2X4 inhibitor), and A438079 (P2X7 inhibitor) alone or in combination for 15 min before 1000 µM ATP stimulation at the concentrations indicated (*n* = 3 separate experiments using different MC cultures). Statistical analysis showed no significant difference between the untreated controls and IL-33-primed cells. Data are presented as mean ± SEM. Statistical differences are indicated; * *p* < 0.05 (one-way ANOVA with Sidak’s post hoc test).

As the use of P2X7 inhibitors highlighted the importance of P2X7 in ATP-mediated MC activation, we sought to investigate whether P2X1 and P2X4 receptors might also play a role in MC degranulation. MCs were pre-treated with 5 ng/mL IL-33 for 24 h, and prior to stimulation with ATP, the P2X1 inhibitor NF449 and P2X4 inhibitor 5BDBD were applied for 15 min (Figure 6A).

Unlike the effect observed using P2X7 inhibitors, P2X1 or P2X4 inhibition showed no reduction in MC degranulation, independent of the ATP concentration used, or IL-33 priming.

These results further suggest that P2X1 and P2X4 are not significantly involved in ATP-mediated MC degranulation.

### 2.7. P2X1 and P2X4 Receptors Affect P2X7 Receptor Function

We have demonstrated that the P2X7, not P2X1 or P2X4, contributes to ATP-mediated MC degranulation and receptor expression and that these activities are modulated by IL-33. However, since IL-33 modulates P2X1 or P2X4 expression and have been shown to modulate P2X7 responses in other cell types [39,40], we investigated whether these receptors could affect ATP-mediated MC degranulation when acting in concert with P2X7 rather than individually. Therefore, MCs were primed with IL-33 and then incubated with P2X1, P2X4, and P2X7 inhibitors (NF449, 5BDBD, A438079), alone or in combination, before being subjected to stimulation with ATP.

As previously observed, the presence of the P2X7 inhibitor, A438079, led to a decrease in MC degranulation, while the use of the P2X1 (NF449) and P2X4 (5BDBD) inhibitors, alone or in combination, had no effect (Figure 6B). However, when the P2X1 or P2X4 inhibitors were mixed with the P2X7 inhibitor, MC degranulation was minimal in both the untreated and IL-33-primed cells. In particular, the combination of the P2X1 and P2X7 inhibitors resulted in the complete inhibition of MC degranulation. However, we did not observe any significant differences between the untreated and IL-33-primed cells. Thus, our findings suggest that the P2X1 and P2X4 receptors exerted efflux and influx influences on P2X7 receptor activation, irrespective of the IL-33 priming concentration used.

## 3. Discussion

Our study expands the current knowledge on the role of P2X receptors in human MC activation and their contribution to, and regulation of, ATP-mediated degranulation through describing experiments involving a IL-33 priming model. We have shown that high ATP concentrations lead to MC degranulation, which is in line with the results published in a previous study [16] wherein the stimulation of human LAD2 cells induced degranulation at similar levels. Our results also align with the findings obtained by Gao et al. [41], who showed that while ADP can potentiate degranulation in the LAD2 cell line, in the presence of complement or antigenic stimulation, ADP alone has no effect.

IL-33 exerts an important influence on a broad range of biological processes related to inflammatory conditions such as promoting the release of pro-inflammatory cytokines and chemokines [42]. Our results demonstrate the influence of IL-33 in potentiating ATP-mediated MC activities, namely degranulation, IL-8 release, and pERK1/2 signalling. These results are consistent with those of previous studies that have reported increased MC activation after IL-33 priming upon IgE or complement stimulation [24,43]. In contrast, the simultaneous administration of IL-33 and ATP showed no additive effect on mouse BMMC degranulation, suggesting the importance of sequential exposure [30]. IL-33 priming induces IL-8 cytokine production at lower concentrations of ATP compared to degranulation (10 μΜ versus 100 μΜ, respectively). We speculate that this discrepancy is due to two separate causes. First, IL-33 produces an increased release of IL-8, which can be boosted by activation [44], as also observed in our model using both IgE/anti-IgE and ATP.

Second, MC IL-8 release can occur independently of degranulation [45] or the need for a different dose, as observed when using IgE/anti-IgE, Substance P, or other stimuli [46,47].

Consequently, these results suggest that even low ATP concentrations can initiate localized inflammatory conditions, as IL-8 acts as a potent chemotactic agent for granulocytes and other immune and non-immune cells [48]. Furthermore, in neutrophils, IL-8 promotes the direct activation and release of neutrophil extracellular traps [49] and serine proteases [50], which could contribute to inflammatory responses. In fact, IL-8 modulation is known to play a role in diseases such as chronic obstructive pulmonary disease (COPD), cystic fibrosis (CF), and COVID-19 [51,52,53].

While IL-33 priming significantly modulated ATP-mediated MC activation, it did not affect ADP-mediated stimulation. Previously, ADP has been found to enhance antigen-mediated MC degranulation in rat MCs through the activation of the P2Y13 receptor, while exposing P2Y1 to ADP alone has been shown to lead to intracellular calcium mobilization [36]. It therefore appears that ADP only acts synergistically when MCs have been previously exposed to direct activation, but ADP cannot induce MC degranulation in human MCs alone. Also, in combination with IL-33, the effect of ADP on MC degranulation is minimal.

TSLP and IL-33 are alarmins released by damaged or necrotic epithelial and endothelial cells, affecting MC functionality in inflammatory conditions [24]. Our results therefore show that the effects of IL-33 and TSLP vary in their specific modulatory activities on ATP-mediated MC activation.

While IL-33 priming increased the membrane expression of the P2X1 and P2X4 receptors, as well as, to a certain extent, that of P2X7, we did not observe any significant increase in transcription. These results contrast with the ones of Jordan et al. [30], who reported that IL-33 upregulated P2X4 and P2X7 transcriptional expression in mouse BMMCs. These results not only highlight major differences between human and mouse MC systems but also suggest that IL-33 modulates the membrane expression of P2X receptors post-transcriptionally, possibly affecting their trafficking to the cell membrane.

The use of orthosteric and allosteric P2X7 receptor inhibitors proved that ATP-mediated MC degranulation occurs mainly through P2X7 engagement, as their use significantly reduced MC degranulation. These results further establish P2X7 as the main receptor for ATP-mediated degranulation in humans, confirming previous findings obtained using MCs from different tissues and species or cell lines such as the LAD2 cell line, mouse BMMCs, mouse peritoneal and meningeal MCs, mouse mastocytoma cells (P815), and MCs from rat spinal cords [16,17,30,54,55,56]. The use of two different P2X7 inhibitor classes also revealed variable effects of IL-33 priming, possibly elicited by conformational changes, the modulation of receptor crosstalk, or P2X7 trafficking. Further studies are needed to elucidate the underlying mechanisms of the IL-33-mediated modulation of P2X7 receptor function.

The activation of P2XRs leads to increased downstream signalling through a wide array of pathways, namely the ERK1/2, STAT3, NF-κB, Sarcoma Tyrosine Kinase, Protein Kinase C, MAPK, and Phosphoinositide 3-Kinase pathways [57,58,59]. We demonstrated that different ATP concentrations promote signalling through the ERK1/2 pathway. Of note, the observed increased activation using ATP concentrations less than 100 μM suggests the involvement of P2XRs other than P2X7, since concentrations over 100–300 μM are required to elicit its activation [16]. When investigating the effect of IL-33 priming on pERK1/2 signalling, we showed that only low IL-33 concentrations potentiate the signalling effect.

Allosteric inhibitors effectively suppressed IL-33-primed ATP-mediated MC degranulation, while orthosteric inhibitors did not significantly reduce ATP-mediated degranulation through the P2X7 receptor. While allosteric inhibitors exert their function outside of ATP binding sites by interfering with ATP binding via conformational receptor changes [60,61], orthosteric inhibitors need to occupy all three ATP binding pockets on the P2X7 receptor to produce full receptor inhibition [59,62], as ATP occupancy fully stabilizes the P2X7 open state [63]. Since IL-33 produces a modulation in P2X7 receptor membrane expression, this may result in the need for increased concentrations of orthosteric inhibitors to fully block all available receptors, ultimately failing to fully inhibit ATP activation at equivalent doses. Conversely, allosteric inhibitors require only one molecule to fully inhibit P2X7 activation, and could therefore be less affected by IL-33 receptor modulation compared to orthosteric inhibitors. The use of the orthosteric A438079 P2X7 inhibitor significantly reduced P2X7-mediated pERK1/2 signalling, regardless of IL-33 priming, unlike the effect observed in MC degranulation, hence suggesting that ATP-mediated degranulation can be both dependent and independent of ERK1/2 activation, depending on the circumstances and the activation of other receptors such as P2X1 and P2X4.

Furthermore, we demonstrated that P2X1 and P2X4 have a potential effect on ATP-dependent P2X7 receptor activation, since the use of P2X7 inhibitors in combination with either P2X1 or P2X4 inhibitors further inhibited ATP-mediated degranulation, with the highest inhibition being achieved when using P2X1 and P2X7 inhibitors simultaneously. However, priming with IL-33 did not substantially modify the observed inhibitory responses, suggesting that IL-33 preferentially modulates the P2X7 receptor activities investigated. These findings further outline the possible importance of P2X1 and P2X4 in MC activation and in other tissues and species, since other authors have demonstrated their contribution in calcium influx and the P2X4-mediated enhancement of IgE-mediated degranulation in BMMCS [16,17,64].

However, it is important to acknowledge the limitations of our study. Donor heterogeneity in the ATP responses among the different MC cultures posed technical challenges, as did the limited number of cells generated per each cell culture. Furthermore, MCs cultured from blood haematopoietic progenitors may differ in receptor expression, granule composition, phenotype, and sensitivity to stimulation compared to tissue MCs. However, tissue MCs themselves exhibit high tissue-specific morphological and functional heterogeneity [65].

In conclusion, our results reveal the distinctive modulatory properties of IL-33 priming on ATP-mediated MC activation, degranulation, intracellular signalling, and cytokine release in human primary MCs, corroborating the data observed in other MC models. Additionally, our results underscore the significant role of the P2X7 receptor in modulating MC activities, hinting at its possible role in inflammation.

## 4. Materials and Methods

### 4.1. Generation of Human Blood-Derived Mast Cells

Human peripheral blood mononuclear cells (PBMCs) were isolated from leukocyte cones as previously described [66,67]. Briefly, leukocyte cones were obtained from the National Health Service Blood and Transplant blood bank (Manchester, UK) from 58 anonymous healthy volunteers who gave informed consent for their donated samples to be used for research purposes, as per the protocol approved by the University of Manchester Research Ethics Committee (UREC ref 2018-2696-5711). PBMCs were isolated using Ficoll-Paque (GE healthcare, Amersham, UK) density gradient centrifugation, and CD117+ progenitors were isolated by positive magnetic selection by using the MACS CD117 microbead kit (Miltenyi Biotec, Bisley, UK) following the manufacturer’s instructions.

Isolated PBMCs were cultured for 4 weeks in media supplemented with 0.5% BSA Fraction V (Gibco, New York, NY, USA), 1% of Insulin transferrin (Gibco, New York, NY, USA), and 100 μg/mL Penicillin–Streptomycin (Sigma-Aldrich, Gillingham, UK) containing 100 ng/mL of human Stem Cell Factor (GenScript, Oxford, UK), 50 ng/mL of IL-6 (GenScript, Oxford, UK), and 100 ng/mL of IL-3 (PeproTech, Cranbury, NJ, USA). At the end of week 4, the culture media were progressively substituted with media devoid of IL-3. After 8 weeks, the cells were tested for functional maturity using IgE/anti-IgE stimulation, and the activation markers CD63 (cloneH5C6, BioLegend, San Diego, CA, USA) and CD107a (clone H4A3, BioLegend, San Diego, CA, USA) were used as a proxy for degranulation and measured via flow cytometry.

### 4.2. Flow Cytometric Analysis of Mast Cell Degranulation

Human MCs were seeded at a 10^6^ cells/mL concentration and treated for 24 h with either 5 ng/mL or 50 ng/mL of IL-33 (PeproTech, Cranbury, NJ, USA), or with 5 ng/mL TSLP (PeproTech, Cranbury, NJ, USA). The MCs were then washed and subsequently stimulated with ATP (ThermoFisher, Vilnius, Lithuania) at concentrations of 10 µM, 100 µM, and 1000 µM, or with ADP (Sigma-Aldrich, Gillingham, UK) at concentrations of 10 µM, 100 µM, and 1000 µM, for a period of 1 h without IL-33 pre-treatment. For the degranulation assay using IgE/anti-IgE stimulation, the cells were treated overnight with 1 µg/mL of human IgE (Merck, Gillingham, UK) and stimulated with 1 µg/mL of goat anti-human anti-IgE (SeraCare, Milford, MA, USA) for 1 h.

For the P2X inhibition experiments, 5 μM of either NF449 (Tocris, Abingdon, UK), 5-BDBD (Sigma-Aldrich, Gillingham, UK), A438079, A839977, AZ-10606120, or AZ-11645373 (Alomone labs, Jerusalem, Israel) inhibitors were dispensed into IL-33-pre-treated cells and washed cells for 15 min, and the cells were subsequently stimulated with ATP.

The cells were then washed in FACS buffer (PBS, 2% FCS, 2 mM EDTA) and incubated with CD63, CD107a, and CD117 (clone A3C6E2) antibodies, with 5 µg/mL of Fc receptor blocking reagent being added (BioLegend, San Diego, CA, USA), along with fluorescence minus one (FMO) as a control. The cells were then stained with Live/Dead™ blue reagent (Thermo Fisher, Eugene, OR, USA), washed using PBS, and fixed with 4% formaldehyde solution (Thermo Fisher, Eugene, OR, USA). The cells were analysed on an LSR-II flow cytometer and subjected to a subsequent analysis conducted using FlowJo^®^ software (BD Biosciences, Wokingham, UK).

### 4.3. Flow Cytometric Analysis of P2X Expression

Human MCs were seeded at a concentration of 10^6^ cells/mL and treated for 24 h with 5 ng/mL or 50 ng/mL IL-33. The cells were then washed in FACS buffer (PBS, 2% *v*/*v* FCS, 2 mM EDTA) and incubated with either anti-P2X1 (1 mg/mL) (Cat# APR-022; isotype rabbit IgG1), anti-P2X4 (1 mg/mL) (Cat# APR-024; isotype rabbit IgG1), or anti-P2X7 (1 mg/mL) (Cat# APR-008; isotype rabbit IgG1; Alomone labs, Jerusalem, Israel) primary antibodies, together with 5 µg/mL of Fc block. The cells were then washed and incubated with Alexa Fluor 488 secondary antibody (2 mg/mL) (Thermo Fisher, Eugene, USA). The cells were washed with PBS before and after cell staining with live/dead reagent and subsequently fixed with 4% formaldehyde solution. The cells were analysed on a LSR-II flow cytometer and subjected to a subsequent analysis conducted using FlowJo^®^ software version 10.8.1.

### 4.4. Flow Cytometric Analysis of ERK1/2 Phosphorylation

Human MCs were seeded at a 10^6^ cells/mL concentration and treated with either 5 ng/mL or 50 ng/mL IL-33. The cells were washed, rested for 2 h in media devoid of IL-6, and subsequently stained with Live/Dead™ reagent. The cells were then activated for either 0, 1, 5, 10, 15, 30, or 45 min by ATP or BzATP (Sigma-Aldrich, Gillingham, UK) at a concentration of 10 µM or 100 µM. For when the P2X7 inhibitor A438079 was used, the cells were treated with 5 μM of the inhibitor for 15 min prior to stimulation. The reaction was stopped at the appropriate time by adding 1.6% formaldehyde solution diluted in PBS and permeabilised in 100% Methanol. The cells were then washed again and incubated with anti-phospho-ERK1/2 (pERK1/2, clone MILAN8R; isotype mouse IgG1; Thermo Fisher, Eugene, USA), together with Fc receptor blocking reagent. The cells were analysed on a LSR-II flow cytometer and subjected to a subsequent analysis conducted using FlowJo^®^ software version 10.8.1.

### 4.5. Cytokine Release Measurement Using Cytometric Bead Array (CBA)

Measurements of the number of released IL-8 cytokines were performed using CBA on supernatants obtained from human MCs seeded at a 10^6^ cells/mL concentration and treated for 24 h with 5 ng/mL or 50 ng/mL of IL-33. The cells were then washed and subsequently stimulated with ATP concentrations of 10 µM, 100 µM, and 1000 µM or with ADP 1000 µM for 8 h. For IgE/anti-IgE stimulation, the cells were treated overnight with 1 µg/mL of human IgE and stimulated with 1 µg/mL of goat anti-human anti-IgE for 1 h. For CBA, the human IL-8 Flex Set Kit was used according to the manufacturer’s instructions (BD Biosciences, Wokingham, UK). An analysis was carried out using a BD FACSVERSE™ cytometer, and the cells were analysed using FCAP Array™ v3.0.1 Software (BD Biosciences, Wokingham, UK).

### 4.6. RNA Sequencing

MCs (10^6^ cells/mL) from individual donors were treated with IL-33 50 ng/mL or left untreated for 24 h at 37 °C and 5% CO_2_ in culture media devoid of IL-6. After treatment, mRNA was extracted using the RNA Easy Micro Kit (Qiagen, Tokyo, Japan) according to the protocol supplied. Extracted RNA was analysed using an Illumina HiSeq4000 sequencer (Illumina, San Diego, CA, USA) [68]. Data pre-processing and alignment were carried out by the Genomics Core Facility of the University of Manchester. The aligned reads were then further analysed by the Bioinformatics Core Facility of the University of Manchester, who used the R DESeq2 package [69], with significance set at an adjusted *p* value of <1e-04 (false discovery rate Benjamini–Hochberg method) for differentially expressed genes.

### 4.7. Statistical Analysis

Data were analysed using a one-way ANOVA with Šídák’s post hoc comparison tests when studying the responses to different ATP/ADP concentrations or IL-33 pre-treatment. A two-way ANOVA with Šídák’s post hoc test was used when studying the combined effect of cytokine pre-incubation and ATP/ADP responses, as indicated. Significance for the ANOVAs was set at *p* < 0.05. The differential expression of RNA sequencing data was calculated using the Wald test, and significance was set at a false discovery rate adjusted *p* of < 1e-04. All data were processed and analysed using GraphPad prism software v9, and significant differences are indicated as follows: * *p* < 0.05, ** *p* < 0.01 *** *p* < 0.01, and **** *p* < 0.0001. Data are presented as mean ± SEM of independent experiments using individual MC cultures.

## Data Availability

Data are contained within the article or Supplementary Materials.

## Supplementary Materials

The supporting information can be downloaded at: https://www.mdpi.com/article/10.3390/ijms25031730/s1.

## Abbreviations

ATP	Adenosine triphosphate
ADP	Adenosine diphosphate
BMMC	Bone marrow mast cell
GM-CSF	Granulocyte–macrophage colony-stimulating factor
GMFI	Geometric mean fluorescence intensity
IL-33	Interleukin-33
MC	Mast cell
PBMC	Peripheral blood mononuclear cell
P2XR	P2X receptor
TSLP	Thymic stromal lymphopoietin
