# Supplementary material for: P2X7 Receptor-Induced Human Mast Cell Degranulation Is Enhanced by Interleukin 33
Source: Int J Mol Sci. 2024 Jan 31;25(3):1730. doi: 10.3390/ijms25031730 (PMC10855801; doi:10.3390/ijms25031730)
Supplement: Supplementary file 1 [file ijms-25-01730-s001.zip › ijms-2808022-supplementary.pdf]

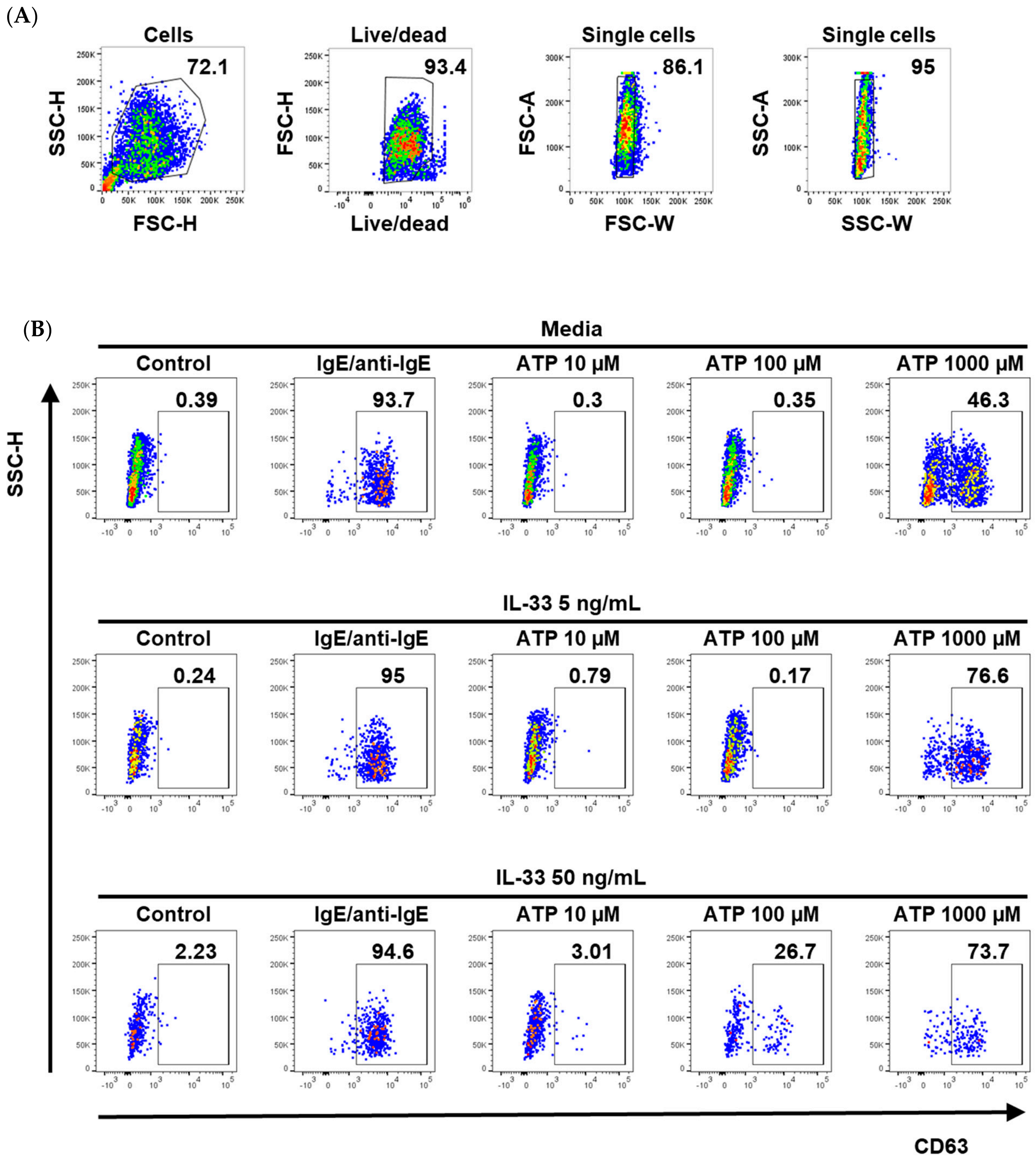

Figure S1. Example of the gating strategy used when assessing degranulation of blood-derived mature human mast cells.

(A) The MC population was selected according to size and granularity using forwards and side scatter. Dead cells were excluded using Live/dead discrimination, with single cells selected according to area and width parameters.

(B) Gating strategy showing the example of MCs degranulation based on CD63 externalisation upon IL-33 treatment and ATP or IgE/anti-IgE stimulation.

(**A**)

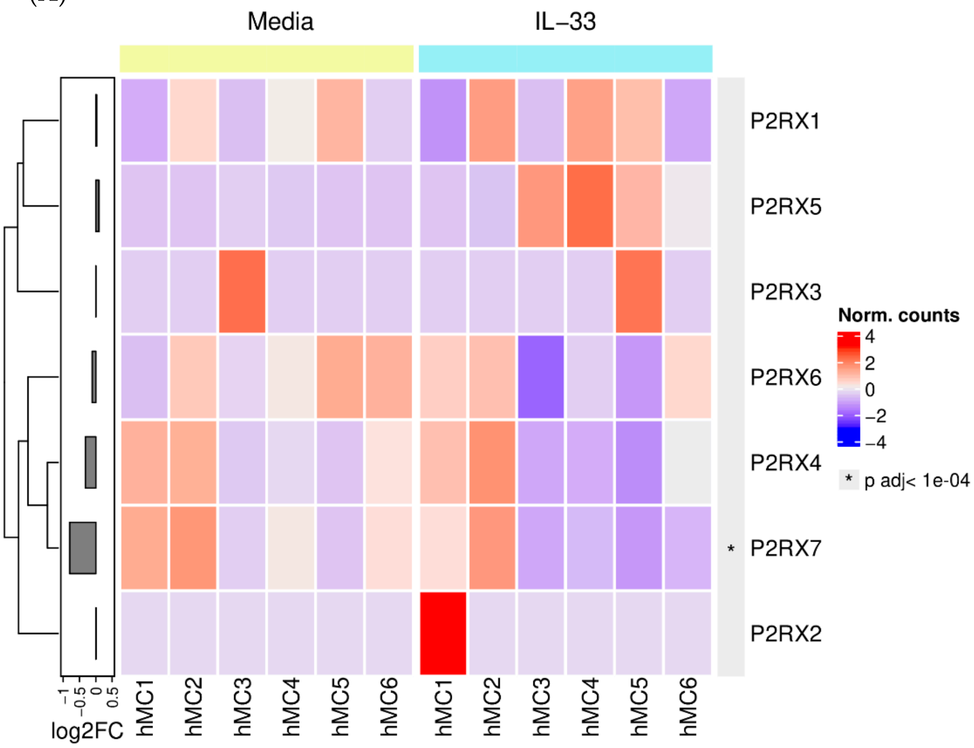

(**B**)

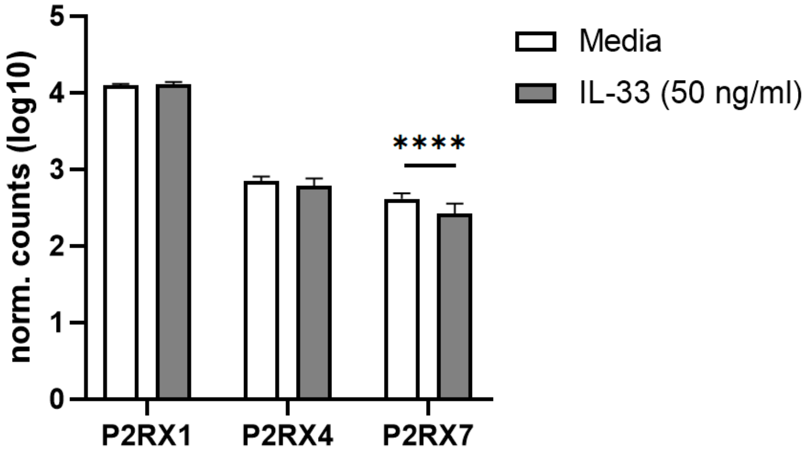

Figure S2. Changes in P2X receptor gene expression upon IL-33 priming in mast cells.
(A) The results for n=6 MC donors were visualised as log2fold change compared to media (left) and z-scores of normalized counts of selected P2XR genes were sorted by treatment group and Euclidean distance and plotted using the R ComplexHeatmap package.
(B) Normalized counts of P2X1, P2X4 and P2X7 were calculated using the R DESeq2 package.
Data are mean ± SEM of n=3 separate experiments from individual MC cultures. Statistical differences are indicated, **** p<0.0001 (Wald test).

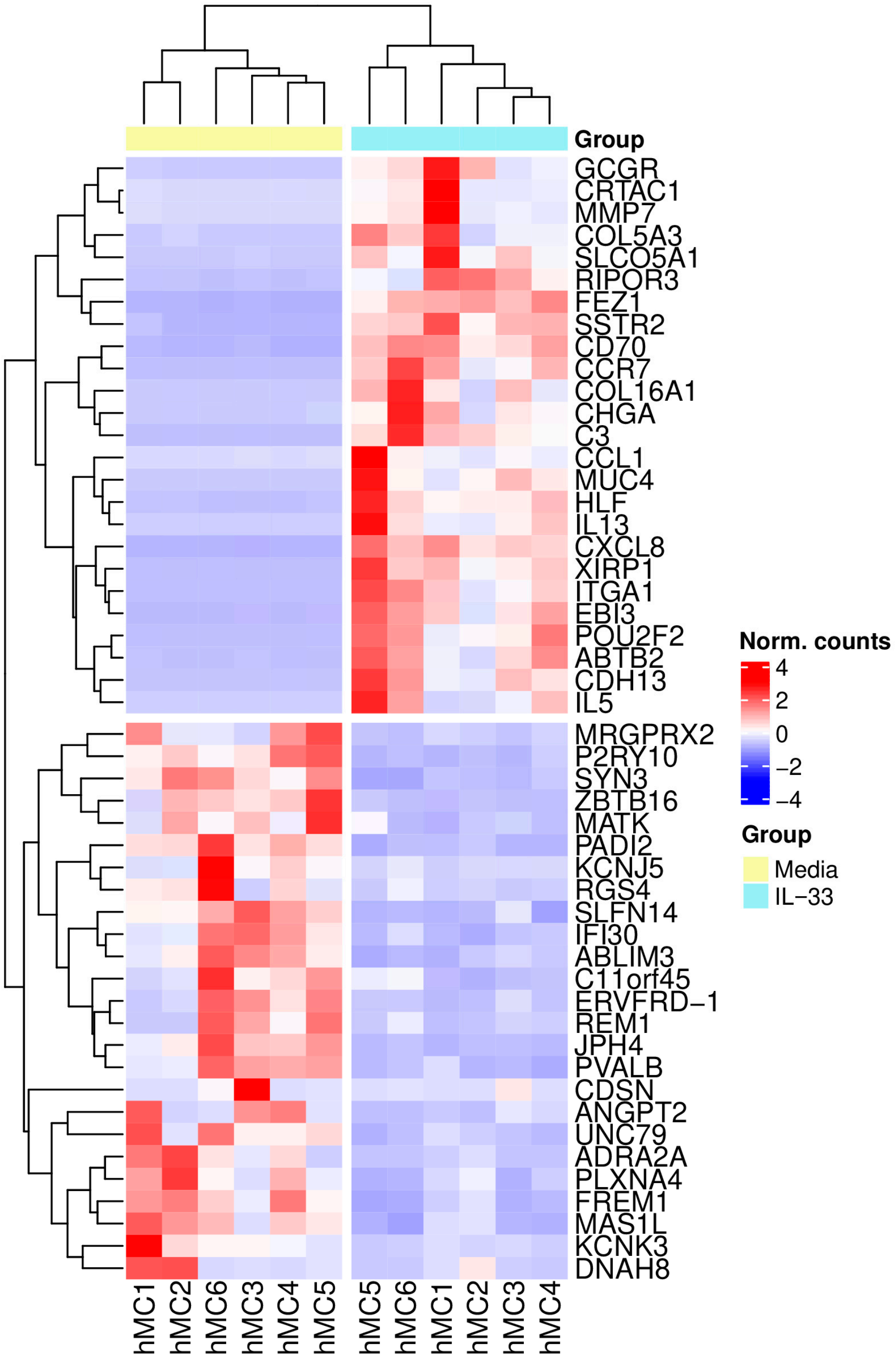

Figure S3. Mast cell gene expression changes in response to 24-hour IL-33 priming.
Top high and lowly expressed 50 protein-coding genes (log2 fold change of ≥ 2 or ≤-2, adjusted p-value < 1e-04) visualised as a z-score values. The results for n=6 MC donors were visualised as a heatmap generated using the R pheatmap package and sorted by Euclidean distance.
